# Hospital Meetings

**Published:** 1896-06-06

**Authors:** 


### HOSPITAL MEETINGS.

### MIDDLESEX HOSPITAL QUARTERLY COURT.

A Quarterly Court of Governors was held at the Middlesex Hospital on May 28th, Mr. E. W. Nunn in the chair. There were also present: The Right Hon. Sir R. Thompson, K.C.B., Sir Arthur Townley Watson, Bart., Mr. Francis Hoare (treasurer), Lieutenant-Colonel T. Lambert Mears, L.L.D., and the Rev. Canon Acheson. Satisfactory progress was reported of the new convalescent home at Clacton, which will be opened on July 1st. The Duke and Duchess of York have consented to inaugurate the fête which will be held in the hospital grounds on that day to celebrate the event, and will receive purses of £5 5s. and over. The convalescent home is estimated to cost, when complete, the sum of £30,000, of which two-thirds have been paid from money at the disposal of the Samaritan Fund, aided by an advance of £16,000 on mortgage from the Spicer trustees, and a loan of £2,000 from the bankers. There is a pressing need now for liberal contributions to free the building from all debt. The Middlesex Hospital has to regret the loss by death of two generous benefactors during the past quarter, Baron de Hirsch, vice-president since 1893, and Mr. William Debenham, for nineteen years a member of the Weekly Board. The Right Hon. Viscount Gort has been elected to the vacant office of vice-president. Mr. George Lawson, F.R.C.S., senior surgeon, has retired from the active staff, of which he has been a member for 33 years, and has been duly appointed consulting surgeon. To the vacant office of surgeon Mr. Pearce Gould, F.R.C.S., M.S., has been elected.

### HOSPITAL FOR CONSUMPTION, BROMPTON.

The annual Court of Governors was held at the hospital on the 28th ult., Mr. T. P. Beckwith (chairman of the committee of management) presiding. The report, read by the Secretary (Mr. W. H. Theobald), showed that 1,492 in patients were admitted during 1895, the average stay of each patient being 68 days. The daily average number of beds occupied was 224. Thirteen thousand three hundred and ninety-eight new cases had received medical treatment and medicines as out-patients, while 72,646 attendances were registered. The annual subscriptions had decreased, but donations and legacies were larger than in the previous year. The receipts during the year amounted to £42,725, included in which amount was £19,256 received in legacies. The total expenditure was £30,033, and £1,147 was temporarily invested. The first portion of the exterior stonework of the old building had been restored, and the second portion was progressing satisfactorily. The electric lighting had been installed in both the old and new buildings. The Chairman, during the course of the proceedings, mentioned that the committee had acquired buildings near the hospital which they hoped, as soon as the present tenants' leases expired, to use in providing accommodation for their nursing staff.

### CHILDREN'S FREE HOLIDAY HOME AND ORPHANS' AID FUND.

The annual meeting of this society was held at Exeter Hall, under the presidency of the Ven. Archdeacon Sinclair, on the 28th ult. The objects of the society (which has been in existence for about eight years) as set forth in the report are to provide poor children, through the Board schools and other charitable agencies, with a free holiday at the seaside or with a free ticket for food. From the same source we find that in the year ended April 30th, 1896, 353 children were sent for a week each to the "home" at Southend, 210 were sent for a day at the seaside and provided with dinner and tea, and 10,000 free tickets for breakfast, dinner, or tea were issued. According to the balance-sheet we find that no less than 58 per cent. of the income is spent on management, while only 42 per cent. directly benefits the children for whom it was intended. Thus the expenditure on provisions, coal, furniture, railway fares, clothing, relief to widows, grants for meals (that is to say on the children themselves) is only £349, whilst the amount spent on what may be called office expenses is £486. Putting it in another way, charitable persons who read in the leaflets issued by the society that 10s. will provide 60 children with dinner or 120 with breakfast, and forthwith put their hands in their pockets and send half-a-sovereign to the society, should observe that 5s. 10d. of that half-sovereign will be swallowed up in management, and the balance will only give 25 children a dinner or 50 a breakfast. An item in the balance-sheet shows the cost of the ladies' and children's street collections. £367 was obtained through street collections in 1895-6, but the cost of these collections was £105, or nearly 30 per cent. Thus only about one-third of every sixpence so given reaches the children. According to the balance-sheet 1¾d., out of that sixpence goes to pay the expenses of collection, a further 2¼d. is wanted for office expenses, and only the residue—twopence—directly benefits the children.

### THE HOSPITAL FOR SICK CHILDREN.

The annual meeting of the Great Ormond Street Children's Hospital was held at the hospital on the 29th ult., Viscount Gort in the chair. The committee in their report drew the special attention of the governors to the falling off in annual subscriptions, only £2,858 having been received from this source in 1895, against £3,122 in 1894, or a decrease of £264. This decrease we hope is only temporary, as so excellent an institution as this, the largest and the oldest children's hospital in the kingdom, ought surely never to be overlooked by the charitable. Mr. Arthur Lucas, the chairman of the committee, in moving the adoption of the report, said that though the expenditure of the year 1895 had been met by the income, yet 1896 had not been so satisfactory. At the time of the annual meeting the committee had money enough to go on till October next, but in that month, unless they received further funds, they would either have to sell out the very small amount of stock they held or run into debt. Personally he was very much against the latter course, because that hospital had never run up a debt for maintenance.

